# People at Risk of Influenza Pandemics: The Evolution of Perception and Behavior

**DOI:** 10.1371/journal.pone.0144868

**Published:** 2015-12-14

**Authors:** Jianhua Xu, Zongchao Peng

**Affiliations:** 1 College of Environmental Sciences and Engineering, Peking University, Beijing, P. R. China; 2 Center for Crisis Management Research, School of Public Policy and Management, Tsinghua University, Beijing, P. R. China; University of Hong Kong, HONG KONG

## Abstract

Influenza pandemics can severely impact human health and society. Understanding public perception and behavior toward influenza pandemics is important for minimizing the effects of such events. Public perception and behavior are expected to change over the course of an influenza pandemic, but this idea has received little attention in previous studies. Our study aimed to understand the dynamics of public perception and behavior over the course of the 2009 H1N1 influenza pandemic. Three consecutive cross-sectional surveys were administered among Beijing residents with random-digit dialing techniques in March 2008 and August and November 2009. Effective samples of 507, 508 and 1006 respondents were interviewed in each of the three surveys, respectively. The mean scores of risk perception were low to moderate across the three surveys. The perceived risk of infection of *self* was significantly lower than that of the *community*, revealing an optimistic bias. Longitudinally, the perceived risk of contracting H1N1 increased, whereas the perceived risk of being unable to obtain medicine and medical care once influenza permeated the community first increased and then decreased. Responsive actions toward influenza varied. Most respondents took actions that required little extra effort, such as ventilating rooms; these actions did not change over time. Comparatively, a smaller number of respondents took actions for coping with influenza, such as vaccination; however, these actions were taken by an increasing number of respondents over time. The association between risk perception and behavior was unstable. Positive, insignificant, and negative associations were obtained in the three surveys. In conclusion, the evolving patterns of risk perception and responsive behavior over the course of an influenza pandemic are sensitive to how risk and behavior are defined and scoped.

## Introduction

Influenza pandemics occur irregularly, at intervals of 10–50 years [1], yet they can generate significant impacts on human health and society [2,3]. For instance, the notorious 1918 Spanish flu claimed the lives of approximately 50 million people worldwide [4] and was responsible for a significant increase in poorhouse rates in Sweden [5]. When an influenza pandemic breaks out, almost everyone is at risk [1,6]. Population-based measures can play a crucial role in preventing and containing the spread of influenza. These measures include both pharmaceutical measures, such as vaccination and taking antiviral drugs, and non-pharmaceutical measures, such as social distancing and personal hygiene practices [7,8].

While these measures are well-known to health authorities [8,9], they may not be known by the public. Public information campaigns, which are widely used as policy instruments to influence attitude and behavior in order to achieve desirable social outcomes [10], are needed to inform and mobilize the public in the event of an influenza pandemic [11–13]. Effective communication requires a detailed understanding of how people perceive and respond to the threat of influenza pandemics.

Public perception and behavior are expected to change over the course of an influenza pandemic because information and situations change as the event develops. Patients who routinely refused seasonal flu vaccines keenly inquired about the availability of the vaccine when H1N1 influenza was first detected in 2009; however, when the vaccine was available a few months later, the same patients became reluctant to receive H1N1 flu shots [14]. Over the course of the 2003 severe acute respiratory syndrome (SARS) outbreak, the proportion of Hong Kong residents who adopted personal protective measures increased while their perceived level of risk of contracting the disease decreased [15].

Past experiences regarding public perception and behavior toward influenza pandemics are informative for developing plans to address future pandemics. However, little attention has been paid to the dynamic changes of perceived risk and responsive behavior of the public over the course of an influenza pandemic emergency. A wave of studies was conducted during the 2009 H1N1 influenza pandemic in different countries and in different phases of the emergency [16–26]. These studies were mostly cross-sectional. It is difficult to derive temporal changes in risk perception and behavior from these studies because they differ greatly in measuring risk perception and responsive behavior in terms of scope and scoring [16]. Even when standardized questions were used, risk perception and responsive behavior in the same phase of the emergency varied greatly across countries [27].

We attempted to contribute in this issue with three consecutive surveys administered to Beijing residents in China before and during the 2009 H1N1 influenza pandemic by answering the question: How did the perceived risk and responsive behavior of the public evolve over the course of the 2009 H1N1 influenza pandemic emergency?

## Analytical Framework

Risk is often defined as the probability of a risk *target* losing something of value [28], the basic constructs of which are *uncertainty* and *undesired consequences* [29]. In our context, risk *targets* can be self, immediate family members, and the community, and *undesired consequences* cover both direct effects, which include contracting the disease, and indirect effects, which include what might befall people once influenza permeates the community. The inclusion of risk *targets* of different social distances allows us to examine whether “optimistic bias” exists in perceiving the risk of contracting H1N1 given that previous research shows that people tend to perceive themselves to be less at risk than others [30].

Risks posed by an influenza pandemic can be defined with different combinations of risk *targets* and *undesired consequences*. Thus, it is unsurprising that risks discussed in studies conducted during the 2009 H1N1 influenza were defined somewhat differently [16]. However, in discussing risk perception with regard to influenza pandemics, scholars use the phrase “perceived risk” as though it is a standard term without paying much attention to how risk is defined and measured. In examining the evolution of perceived risk over the course of the 2009 H1N1 influenza pandemic emergency, we examined how results may vary with different measurements of perceived risk.

To mitigate the risk posed by influenza pandemics, the public can take a variety of measures. They can reduce exposure by practicing social distancing and taking personal protection and hygiene measures, they can induce immunity through virus-specific vaccination, and they can mitigate the impacts by stockpiling antivirals. Each measure has its benefits and limitations, and it is generally agreed that multiple measures should be taken simultaneously to mitigate the impact of an influenza pandemic [9]. The foregoing measures are all covered in our study. These measures differ in cost, with some requiring little extra effort, such as ventilating rooms and cough etiquette, whereas others carry a cost, such as purchasing antivirals and seasonal flu shots. In examining the evolution of responsive behavior over the course of the 2009 H1N1 influenza pandemic emergency, we examined how the evolutionary pattern of these different measures may differ.

To explain and predict preventive health behaviors, scholars have put great effort into unraveling individuals’ decision-making processes. Risk perception, defined as the subjective judgment of a risky situation or event [31], is assumed to play a central role in shaping health-related behaviors [32–35]. When reviewing the wave of studies conducted in different countries during the 2009 H1N1 influenza pandemic, it was found that studies that examined the relationship between behavior and risk perception all reported a positive association [16]. Following suit, we hypothesized that the level of perceived risk would also be positively associated with responsive behavior over the course of the H1N1 influenza emergency among Beijing residents in China.

## Case Description

Insights into the evolution of risk perception and responsive behavior over the course of an influenza pandemic would be discounted without any understanding of the context in which the perception and behavior occurred. H1N1 influenza emerged from Mexico and the United States in late March and early April 2009 and then spread globally [36]. The first case confirmed in China was on May 1 in Hong Kong and was imported from Mexico [37]. On the mainland, the first three cases were confirmed on May 11–14, with two imported from the United States and one from Canada [38]. Subsequently, the influenza pandemic broke out in late 2009 and then gradually tailed off in 2010. In this event, most of the cases had mild symptoms, and the proportion of critical or fatal cases was small [39].

This influenza pandemic was not unexpected. In the years before the outbreak of the 2009 H1N1 influenza, there was heightened concern for an influenza pandemic because the essential prerequisites for such an event were almost ready [40]. Many countries developed preparedness plans for an impending influenza pandemic. China developed its preparedness and contingency plan for such pandemics in 2005, following the guidance of the WHO [41], and they put great effort into training professionals for public health emergencies and enhancing the capacity to produce vaccines and antivirals [42]. Meanwhile, catalyzed by 2003’s SARS epidemic, an integrated information system was established to facilitate disease surveillance, detection, reporting, and response [43].

On April 25, the WHO announced that the H1N1 influenza situation constituted “a public health emergency of international concern” and recommended that all countries “intensify surveillance for unusual outbreaks of influenza-like illness and severe pneumonia” [44]. The Chinese government responded swiftly with a series of measures. On April 30, ad hoc working groups and specialty groups were formed to integrate resources from relevant government agencies to prepare for an influenza pandemic. In early May, public health agencies activated the health system contingency plan at all levels. Passengers and cargo were required to be screened at all ports; the Chinese Center for Disease Control and Prevention (Chinese CDC) provided training to lab staff from portal cities on how to detect the H1N1 virus.

Beginning on May 11, the number of new cases and their geographic distributions were updated on a daily basis on the Chinese CDC website. From May 11 to June 11, the number of new cases increased each day from one to approximately a dozen, with most of them being imported. On June 11, the WHO elevated the pandemic alert from Phase 5 to Phase 6. In mid-June, the risk of community-level outbreaks was judged to be increasing, and the focus of surveillance was switched from frontiers to communities. Efforts were then put into preventing spread of the disease at the community level. On June 22, there was an outbreak of H1N1 influenza in an elementary school in a southern city, and a total of 30 cases were confirmed. On July 26, there was an outbreak at a summer camp in Beijing, and a total of 36 cases were confirmed. Meanwhile, clinical trials of the H1N1 vaccine were conducted in July and August.

In late August and early September, the fall semester started, and there was concern about H1N1 influenza in schools. Beginning on September 10, Beijing offered free seasonal flu vaccine shots to students and the elderly over 60. Beginning on September 21, the H1N1 vaccine was administered to all who intended to participate in the parade to celebrate the 60^th^ National Day of the People’s Republic of China. From October to December, there was a sharp increase in the number of new cases, especially in schools, marking the massive outbreak of H1N1 influenza. Because the H1N1 virus had been proven to be mild, the prevention and control strategy was switched to vaccination at that time. In 2010, the event faded off gradually.

## Methods

**Fig 1:**
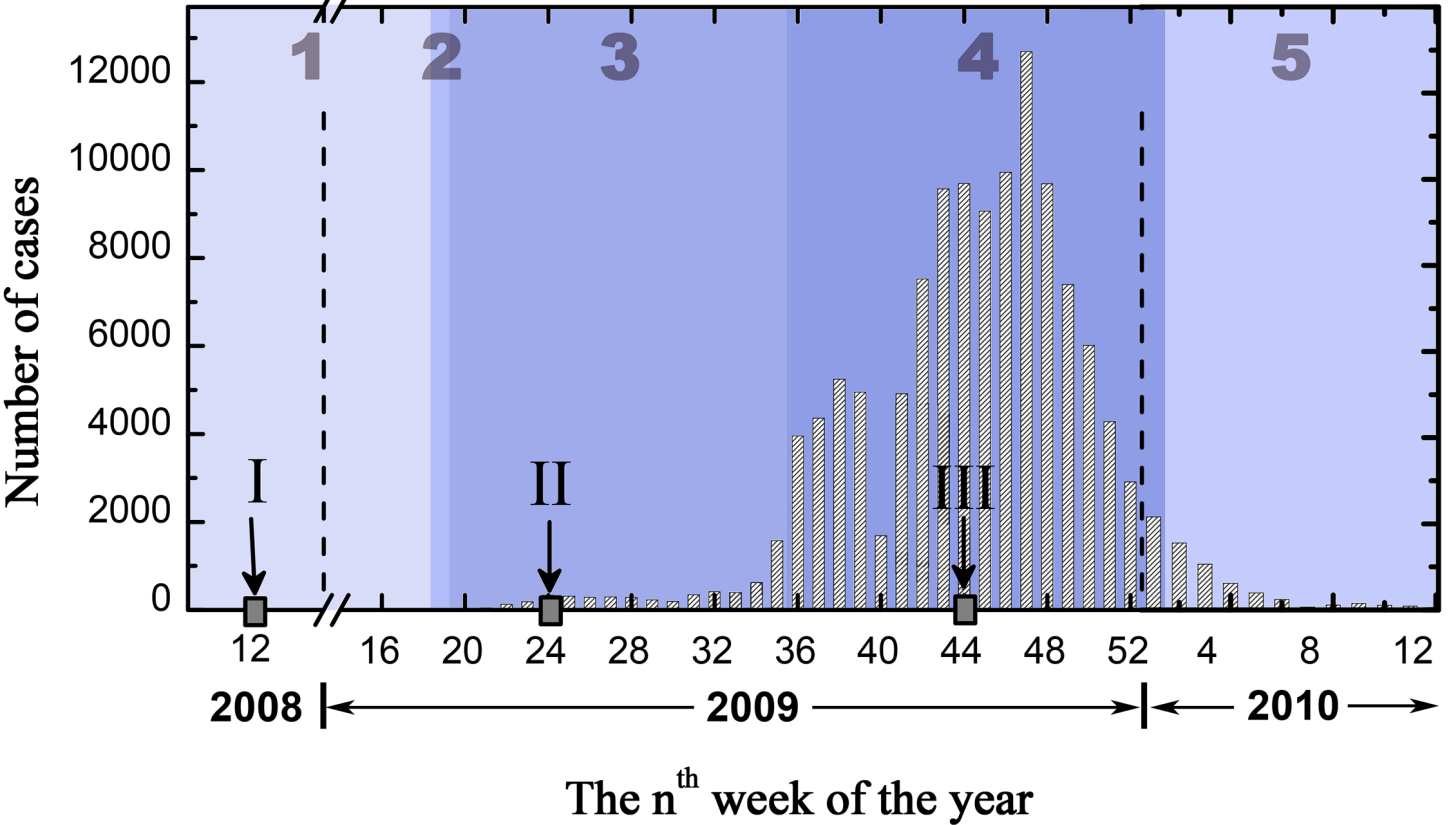
Changes in the reported numbers of H1N1 cases in China over time during the 2009 influenza pandemic, with 1 (preparedness), 2 (early warning), 3 (emerging), 4 (massive outbreak) and 5 (tailing off) indicating the different phases of the event. The three surveys were conducted at points I, II, and III (data source: the Chinese Center for Disease Control and Prevention and China National Influenza Center).

Three cross-sectional surveys were conducted among Beijing residents in March 2008 and August and November 2009. The March 2008 survey was designed to examine the public’s knowledge, attitudes, and behaviors toward an impending influenza pandemic. The August and November 2009 surveys were designed to examine the knowledge, attitudes, and behaviors of the public toward H1N1 influenza. The phases of the flu during which the surveys were conducted are shown in Fig 1, which mainly shows the changes in the reported number of H1N1 cases in China over time.

### Survey

Questions on knowledge, attitudes and behaviors toward an influenza pandemic were covered in the three surveys. Key questions were borrowed from the questionnaire design of the Harvard Opinion Research Program, Harvard School of Public Health, which was used for similar purposes. The accuracy and appropriateness of the questions were evaluated by domain experts from the Chinese CDC, the Institute of Psychology at the Chinese Academy of Sciences, and the Beijing Municipal Institute of Labor Protection, and revisions were made accordingly. The revised questionnaires were then pretested on convenient samples from Tsinghua University, and the wording was fine-tuned based on the results of the pilot tests. The questions on risk perception and responsive behavior were the focus of our study and are detailed below; the remaining questions were briefed but can be found in the surveys’ original format in S1–S3 Files.

Perceived risk was measured in the three surveys with six questions on a five-point Likert scale. Among the six questions, three were about the risk of contracting the disease to self, immediate family members, and the community, and three were about the risk of there being difficulties once influenza permeated the community. The only exception was that in the March 2008 survey, the question on the risk to the community was not asked, and the question “How likely do you think it is that no relatives or friends will be there to take care of you once you contract the disease?” was asked instead. On the five-point Likert scale, “1” represents “not likely,” and “5” represents “very likely.” An additional answer choice, “I don’t know,” was provided for these questions.

The responsive behavior questions covered hygiene and personal protective practices, social distancing measures, information-seeking behavior, and pharmaceutical interventions. In the March 2008 survey, five behavioral items were measured on a five-point Likert scale. The respondents were asked about the degree to which they took the measures, with “1” representing “not at all,” “5” representing “completely,” and “6” representing “I don’t know.” In the August and November surveys, responsive behavior toward H1N1 influenza was measured with twelve items on a binary scale. An example item was “Washing hands with soap and water more often than usual and using alcoholic hand gel more than usual.” For each item, “yes” or “no” was required to be chosen.

Moreover, knowledge on the definition and modes of transmission of influenza pandemics was assessed in the three surveys with multiple-choice questions. Knowledge on the status of the spread of H1N1 influenza was inquired in the two 2009 surveys. Vaccination-related questions and preferred sources of information were asked in the three surveys as well with multiple choice questions. At the end of each questionnaire, demographic and socioeconomic information was asked, including age, gender, education, income and residence.

### Sampling

The surveys were approved by the ad hoc ethics committee of the School of Public Policy and Management (SPPM) at Tsinghua University. This committee is composed of members of the SPPM academic committee. Informed consent from the respondents was obtained orally, as approved by the ethics committee.

The planned samples sizes of 500, 500, and 1000 were determined by the equation *N = Z* ^*2*^ *σ* ^*2*^ */d* ^*2*^ and the budget constraints. The surveys were conducted by the Survey Lab of Tsinghua School of Journalism and Communication, an independent research unit, using random-digit dialing. A computer-assisted telephone interviewing system was used to implement the surveys, and the population included all households in Beijing with landline phones. Phone calls were made in the evenings and on weekends. The person who answered the phone was explained the nature of the survey and asked for their consent. Phone calls were made until the targeted sample size was obtained. In the end, totals of 12989, 10060, and 19906 dials were made, 5076, 4023, and 11301 dials were answered, and the effective samples of 507, 508 and 1006 respondents were obtained. The refusal rates were 90.01%, 87.37%, and 91.10% for the March 2008 and the August and November 2009 surveys, respectively.

### Statistical analysis

SPSS Version 18.0 was used to conduct the statistical analysis. Independent-sample t-test was performed to examine how the public’s perceived risk and responsive behavior evolved over the course of the H1N1 influenza pandemic. Paired t-test was conducted to examine how the public’s perceived risk to themselves, their immediate family members, and the community differed. ANOVA and non-parametric test were performed to examine the differences in perceived risk and responsive behavior among different demographic and socioeconomic groups. Multi-linear regression analysis was used to examine the association between perceived risk and responsive behavior. The demographic characteristics of the respondents in the three surveys are shown in Table 1.

**Table 1:** Demographic information on the respondents.

	March 2008(*n* = 507)	August 2009(*n* = 508)	November 2009(*n* = 1006)
**Gender**			
** Male**	218 (43.00%)	254 (50.00%)	524 (52.09%)
** Female**	284 (56.02%)	254 (50.00%)	482 (47.91%)
** Nonresponse**	5 (0.99%)	0	0
**Age**			
** <18**	0 (0.00%)	100 (19.69%)	41 (4.08%)
** 18–29**	159 (31.36%)	100 (19.69%)	273 (27.14%)
** 30–39**	124 (24.46%)	46 (9.06%)	379 (37.67%)
** 40–49**	86 (16.96%)	62 (12.20%)	138 (13.72%)
** 50–59**	69 (13.61%)	101 (19.88%)	120 (11.93%)
** >60**	55 (10.85%)	99 (19.49%)	55 (5.47%)
** Nonresponse**	14 (2.76%)	0	0
**Highest education level**			
** ≤Elementary school**	29 (5.72%)	17 (3.35%)	25 (2.49%)
** Junior high school**	71 (14.00%)	84 (16.54%)	65 (6.46%)
** Senior high school**	136 (26.82%)	172 (33.86%)	268 (26.64%)
** Associate degree**	—	100 (19.69%)	314 (31.21%)
** 4-year college degree**	225 (44.38%) ^a^	110 (21.65%)	246 (24.45%)
** ≥Master degree**	33 (6.51%)	25 (4.92%)	(8.75%)
** Non-response**	13 (2.56%)	0	0

^a^ “Associate degree” is included here.

## Results

### Risk perception

The perceived level of risk in different phases of the H1N1 influenza pandemic was generally low to moderate (Table 2). Across the three surveys, the average scores for the perceived likelihood of infection were mostly between 2 to 3 (“1” represents “not likely,” and “5” represents “very likely”); similar results were obtained for the perceived likelihood of three undesired consequences once the flu permeated communities (see Q4-Q6 in Table 2).

**Table 2:** Change in public risk perception over time and across risk *targets*.

	Mar. 2008	Aug. 2009	Nov. 2009	Comparison of 2008 and Aug. 2009 surveys	Comparison of Aug. and Nov. 2009 surveys
Q1. How likely do you think it is that you will catch the flu?	1.82	2.34	2.74	*t*(975) = -8.20, *p*<0.01	*t*(1402) = -7.25, *p*<0.01
Q2. How likely do you think it is that your immediate family members will catch the flu?	2.08	2.30	2.69	*t*(970) = -3.50, *p*<0.01	*t*(1396) = -7.69, *p*<0.01
Q3. How likely do you think it is that the flu would permeate your community?	2.00^a^	2.57	3.15	—^a^	*t*(1428) = -11.26, *p*<0.01
Q4. How likely do you think it is that your family would experience financial difficulties once the flu permeated your community?	2.26	2.83	3.10	*t*(971) = -6.80, *p*<0.01	*t*(1426) = -4.12, *p*<0.01
Q5. How likely do you think it is that you would be unable to obtain necessary medicines for preventing or treating the flu once it permeated your community?	2.43	2.75	2.61	*t*(984) = -3.90, *p*<0.01	*t*(1461) = 2.32, *p* = 0.02
Q6. How likely do you think it is that you would be unable to receive necessary medical care once the flu permeated your community?	2.28	2.60	2.44	*t*(992) = -3.98, *p*<0.01	*t*(1469) = 2.57, *p* = 0.01
Composite risk score	2.14	2.59	2.82	*t*(887) = -2.54, *p*<0.01	*t*(1256) = -5.45, *p*<0.01
Comparison of perceived risk to self and perceived risk to immediate family members	*t*(500) = -5.85*p*<0.01	*t*(451) = 1.05 *p* = 0.29	*t*(914) = 1.68, *p* = 0.09	—	—
Comparison of perceived risk to self and perceived risk to the community	—	*t*(446) = -4.87 *p*<0.01	*t*(905) = -14.77 *p*<0.01	—	—
Comparison of perceived risk to immediate family and perceived risk to the community	—	*t*(447) = -5.94 *p*<0.01	*t*(900) = -17.13 *p*<0.01	—	—

^a^ The 2008 survey used the question “How likely do you think it is that no relatives or friends will be there to take care of you once you contract the disease?”

Longitudinally, the scores for each of the six risk perception questions and the composite risk score combining all six were compared between the surveys; the results are shown in the left two columns of Table 2. Perceived risk as measured by the composite risk score increased significantly, from 2.14 to 2.59 (*t*(887) = -2.54, *p*<0.01) and then to 2.82 (*t*(1256) = -5.45, *p*<0.01), over the course of the H1N1 influenza pandemic emergency. The scores for perceived risk of infection to self and immediate family members increased significantly, from 1.82 and 2.08 to 2.34 and 2.30 (*t*(975) = -8.20, *p*<0.01; *t*(970) = -3.50, *p*<0.01) and then to 2.74 and 2.69 (*t*(1402) = -7.25, *p*<0.01; *t*(1396) = -7.69, *p*<0.01). The scores for perceived risk of experiencing financial difficulties once influenza permeated communities increased significantly from 2.26 to 2.83 (*t*(971) = -6.80, *p*<0.01) and then to 3.10 (*t*(1426) = -4.12, *p*<0.01). The scores for perceived risk of being unable to obtain necessary medicines and medical care increased significantly from the preparedness phase (2.43 and 2.28) to the outbreak phase (2.75 and 2.60) (*t*(984) = -3.90, *p*<0.01; *t*(992) = -3.98, *p*<0.01) and then decreased significantly when influenza broke out massively (2.61 and 2.44) (*t*(1461) = 2.32, *p* = 0.02; *t*(1469) = 2.57, *p* = 0.01).

Horizontally, the perceived risks posed to different risk *targets*, i.e., self, immediate family members, and the community, were compared. The results are shown in the three bottom rows of Table 2. The findings from the two surveys during the outbreak of H1N1 influenza were consistent: the scores for perceived risks to “self” (2.34 and 2.74) and “immediate family members” (2.30 and 2.74) were not significantly different (*t*(451) = 1.05 *p* = 0.29; *t*(914) = 1.68, *p* = 0.09), whereas the scores for perceived risk to the “community” (2.57 and 3.15) were significantly higher.

The differences in perceived risk among the different demographic and socioeconomic groups were examined (S1 Table). It was generally found that demographic and socioeconomic factors were not stable predictors of perceived risk, with the results showing positive, negative and insignificant associations, except for self-reported health status and education attainment. The perceived risk of infection was lower among more highly educated groups and among groups with better (self-reported) health status.

### Responsive behavior

#### Public preparedness

The first survey was conducted a year before the outbreak of the 2009 H1N1 influenza, and the public’s behavior reflected their preparedness status. The results showed that 91.1%, 83.4% and 2.4% of the respondents prepared thermometers, medications to treat fever, and Tamiflu at home, respectively, and that 21.5% of the respondents received seasonal flu shots. One hundred ninety-two of the 398 (78.5%) did not receive flu shots; when they were asked why, they expressed that they were strong enough and did not need it.

Most people ventilated their apartments and workplaces, washed their hands with soap and running water, avoided contact with birds, ate thoroughly cooked poultry and eggs, and did not buy uninspected animals and animal products. The survey must be clarified regarding the inclusion of some of the measures that are irrelevant to H1N1 influenza. When the first survey was conducted, H1N1 influenza had not yet broken out. At that time, quite a few places had been hit by the avian flu. Thus, in this survey, some actions that were relevant to avian flu were assessed.

#### Responsive behavior during the H1N1 influenza pandemic

The percentages of respondents who were taking various measures to address the influenza pandemic are shown in Table 3. Actions that required little extra effort were taken by most respondents. For instance, ventilating rooms, washing hands more often, and cough etiquette were practiced by more than 96.0%, 88.0% and 84.0% of the respondents, respectively. Actions that cost some money or demanded extra effort were taken by a comparatively smaller percentage of the respondents. For instance, fewer than 62.0%, 51.0%, and 44.0% of the respondents purchased antivirals, received seasonal flu shots, and stockpiled food and water at home, respectively. The responsive actions that were taken differed somewhat among the different demographic and socioeconomic groups (S2 and S3 Tables). However, the influence of demographic and socioeconomic factors was mostly not stable, which means that positive, negative and insignificant associations between demographic and socioeconomic factors and responsive behavior were found over the course of the 2009 H1N1 influenza.

**Table 3:** Percentage of respondents taking responsive actions over time.

Responsive actions	Aug. 2009	Nov. 2009	Comparison
Hygiene and personal protective practices			
Q1. Ventilating living and working places	96.6%	96.0%	*t*(1510) = 0.59, *p* = 0.55
Q2. Washing hands with soap and water more often than usual and using alcoholic hand gel more than usual	88.9%	88.1%	*t*(1509) = 0.48, *p* = 0.63
Q3. Covering coughs and sneezes with paper tissues, handkerchief, or forearm	84.0%	91.6%	*t*(1510) = -4.46, *p*<0.01
Q4. Purchasing face masks and wearing them in hospitals and public places	50.0%	69.0%	*t*(1510) = -7.32, *p*<0.01
Social distancing measures			
Q5. Staying away from places where many people gather, such as shopping malls	79.2%	88.4%	*t*(1510) = -4.76, *p*<0.01
Q6. Avoiding contact with people from infected areas	86.8%	90.1%	*t*(1510) = -1.93, *p* = 0.05
Q7. Willing to stay at home or be quarantined for 7 days once suspected of or confirmed with H1N1 flu	91.3%	90.6%	*t*(1510) = 0.48, *p* = 0.64
Information-seeking behaviors			
Q8. Talking with doctors or friends about health issues related to H1N1 or swine flu	64.0%	75.4%	*t*(1510) = -4.68, *p*<0.01
Q9. Going to clinics or hospitals upon the onset of flu-like symptoms that are suspicious of H1N1 flu	94.1%	91.6%	*t*(1510) = 1.75, *p*<0.08
Pharmaceutical interventions			
Q10. Purchasing medicines to prevent and treat the flu, such as Tamiflu or Relenza	52.2%	61.1%	*t*(1510) = -3.34, *p*<0.01
Q11. Vaccinating to prevent seasonal flu	36.6%	50.5%	*t*(1510) = -5.17, *p*<0.01
Other measures			
Q12. Stockpiling food and water at home to last for two to four weeks	32.0%	43.2%	*t*(1510) = -4.24, *p*<0.01

Over the course of the H1N1 influenza pandemic, increasing numbers of people took actions to mitigate the risk posed by influenza. Only three types of actions did not change over time: ventilating rooms, washing hands more often, and willingness to conform to isolation and quarantine requirements. These actions had already been taken by a high percentage of the respondents.

#### Risk perception and behavior

The relationship between responsive behavior and risk perception of the public toward influenza pandemics is shown in Table 4. Composite behavior scores and composite risk scores were used. Age, income, gender, education attainment and self-reported health status were used as control variables. Five categories of actions were analyzed: overall responsive behavior, hygiene and personal protective practices, social distancing measures, information-seeking behaviors, and pharmaceutical interventions.

**Table 4:** Coefficients of the variables for predicting responsive behavior.

	Mar. 2008	Aug. 2009	Nov. 2009
Overall responsive behavior			
Self-reported health	0.091**	-0.089*	-0.134***
Gender	0.173***	-0.007	0.037
Education	0.103**	1.294	0.031
Age	0.202***	0.477	0.003
Income	0.013	0.025	0.050
Composite risk score	-0.179***	2.832***	0.034
Hygiene and personal protective practices			
Self-reported health	—	-0.01	-0.098***
Gender	—	0.021	0.056
Education	—	0.034	0.029
Age	—	0.052	-0.035
Income	—	-0.042	0.034
Composite risk score	—	0.068	-0.001
Social distancing measures			
Self-reported health	—	-0.040	-0.028
Gender	—	-0.015	0.045
Education	—	0.137**	0.006
Age	—	0.042	-0.006
Income	—	-0.060	0.077**
Composite risk score	—	0.030	0.075**
Information-seeking behaviors			
Self-reported health	—	-0.138***	-0.065*
Gender	—	0.031	0.059*
Education	—	0.128**	0.004
Age	—	0.087	0.187***
Income	—	0.054	0.065*
Composite risk score	—	0.156***	-0.036
Pharmaceutical interventions			
Self-reported health	—	-0.079	-0.150***
Gender	—	-0.064	-0.013
Education	—	-0.006	0.042
Age	—	-0.019	-0.060
Income	—	0.101*	0.036
Composite risk score	—	0.164***	0.038

^***^<0.01

^**^<0.05

^*^<0.1

Overall responsive behavior was negatively associated with perceived risk in the March 2008 survey, positively associated with perceived risk in the August 2009 survey, and not associated with perceived risk in the November 2009 survey. Hygiene and personal protective practices were not associated with perceived risk. Social distancing measures were positively associated with perceived risk in the November 2009 survey but were not in the August 2009 survey. Public information-seeking behaviors and pharmaceutical interventions were positively associated with perceived risk in the August 2009 survey but not in the November 2009 survey.

## Discussion

Using three consecutive surveys conducted before and during the outbreak of the 2009 H1N1 influenza pandemic, we examined public perception and behavior toward influenza pandemics. We found that the perceived level of risk was low to moderate, the use of responsive actions that required little extra effort was fairly adequate, and the association between responsive behavior and risk perception was not stable.

The perceived risk of infection increased over the course of the emergency. This increase in perceived risk was consistent with the increase in the number of new cases of H1N1 (Fig 1). The perceived risk of being unable to obtain the necessary medicine and medical care increased first and then decreased over time. This may be explained by the characteristics of the flu outbreak. When the H1N1 influenza first broke out, vaccines were not available, and people knew little about the curability of the disease. As time passed, the H1N1 influenza became familiar, a vaccine was available, and the virus was shown to be mild, which made the respondents less worried.

Most respondents took hygiene and personal protective measures that could be practiced in their daily lives even without a public health emergency, such as ventilating rooms and washing hands. Thus, perceived risk had little influence on these behaviors. Pharmaceutical interventions that could incur some cost were taken by a comparatively smaller number of respondents. These measures are where risk communication efforts should be concentrated.

In general, increasing numbers of people took actions to address H1N1 influenza. However, perceived risk was not a stable predictor of responsive behavior. Positive, negative, and non-significant associations between perceived risk and responsive behaviors were found in our study. In most studies conducted during the 2009 H1N1 influenza pandemic, perceived risk was found to be positively associated with responsive behaviors [16]. Two plausible reasons may account for the unstable and inconclusive association in our study. First, perceived risk and behavior are often measured in retrospect. Perceived risk affects behavior, which may in turn affect perceived risk. The measured risk perception and responsive behaviors may have “contaminated” one another. Second, the discrepancy could be related to how risk and behavior are defined and measured. In our study, the associations between perceived risk and different categories of actions were quite different. For instance, hygiene and personal protective practices were not associated with perceived risk, whereas pharmaceutical interventions were.

Finally, *risk* and *behavior* are loose terms. In discussing the patterns of public risk perception and responsive behavior over the course of an influenza pandemic, it is crucial to define the scope of *risk*, *behavior* and risk *targets*. Interpreting the results from existing studies requires attention to the definition and scopes of both risk and behavior as well.

This study provides some evidence on the evolution of public risk perception and responsive behavior toward the H1N1 influenza over time. However, it does have limitations and shortcomings. Perceived risk and responsive behavior were measured in retrospect, which could have made investigating the relationship between responsive behavior and perceived risk problematic.

## Supporting Information

S1 Table Perceived risks among demographic and socioeconomic groups. (DOCX)

S2 Table Responsive behavior among different demographic and socioeconomic groups (2008 survey). (DOCX)

S3 Table Responsive behavior among different demographic and socioeconomic groups (2009 surveys). (DOCX)

S1 File Questionnaire for the March 2008 survey. (DOCX)

S2 File Questionnaire for the August 2009 survey. (DOC)

S3 File Questionnaire for the November 2009 survey. (DOCX)

S4 File Data for the March 2008 survey. (XLS)

S5 File Data for the August 2009 survey. (XLS)

S6 File Data for the November 2009 survey. (XLS)
