# Supplementary material for: People at Risk of Influenza Pandemics: The Evolution of Perception and Behavior
Source: PLoS One. 2015 Dec 14;10(12):e0144868. doi: 10.1371/journal.pone.0144868 (PMC4682843; doi:10.1371/journal.pone.0144868)
Supplement: S1 Table — (DOCX) [file pone.0144868.s001.docx]

**S1 Table. Perceived risks among demographic and socioeconomic groups (“O”: significant difference; “**$\boldsymbol{\times}$**”: no significant difference)**

|  |  | Gender | Age | Education | Self-reported health | Income |
| --- | --- | --- | --- | --- | --- | --- |
| Q1. How likely do you think you are at risk of catching flu? | Mar. 2008 | $\times$ | $\times$ | **O** | **O** | $\times$ |
|  | Aug. 2009 | $\times$ | **O** | **O** | **O** | $\times$ |
|  | Nov. 2009 | $\times$ | **O** | **O** | **O** | $\times$ |
| Q2. How likely do you think your family member or your close friends are at risk of catching the flu? | Mar. 2008 | $\times$ | $\times$ | $\times$ | **O** | $\times$ |
|  | Aug. 2009 | $\times$ | $\times$ | **O** | **O** | **O** |
|  | Nov. 2009 | **O** | **O** | **O** | **O** | **O** |
| Q3. How likely do you think the flu will permeate your community? | Mar. 2008 | — | — | — | — | — |
|  | Aug. 2009 | $\times$ | $\times$ | **O** | **O** | $\times$ |
|  | Nov. 2009 | $\times$ | **O** | **O** | **O** | $\times$ |
| Q4. How likely do you think your family would run financial difficulties once the flu permeates your community? | Mar. 2008 | $\times$ | **O** | **O** | $\times$ | **O** |
|  | Aug. 2009 | **O** | **O** | $\times$ | $\times$ | $\times$ |
|  | Nov. 2009 | **O** | **O** | **O** | $\times$ | $\times$ |
| Q5. How likely do you think you would not be able to get necessary medicines for preventing or treating the flue once the flu permeates your community? | Mar. 2008 | $\times$ | $\times$ | $\times$ | $\times$ | **O** |
|  | Aug. 2009 | **O** | **O** | **O** | $\times$ | $\times$ |
|  | Nov. 2009 | $\times$ | **O** | **O** | $\times$ | **O** |
| Q6. How likely do you think you would not be able to get necessary medical care once the flu permeates your community? | Mar. 2008 | $\times$ | $\times$ | $\times$ | $\times$ | **O** |
|  | Aug. 2009 | $\times$ | $\times$ | $\times$ | $\times$ | $\times$ |
|  | Nov. 2009 | $\times$ | **O** | **O** | $\times$ | **O** |
