# Supplementary material for: People at Risk of Influenza Pandemics: The Evolution of Perception and Behavior
Source: PLoS One. 2015 Dec 14;10(12):e0144868. doi: 10.1371/journal.pone.0144868 (PMC4682843; doi:10.1371/journal.pone.0144868)
Supplement: S2 Table — (DOCX) [file pone.0144868.s002.docx]

**S2 Table. Responsive behavior among different demographic and socioeconomic groups (2008 survey) (“O”: significant difference; “**$\boldsymbol{\times}$**”: no significant difference)**

|  |  | Gender | Age | Education | Self-reported health | Income |
| --- | --- | --- | --- | --- | --- | --- |
| 1. Ventilating living and working places | Mar. 2008 | **O** | $\boldsymbol{\times}$ | $\boldsymbol{\times}$ | $\boldsymbol{\times}$ | $\boldsymbol{\times}$ |
| 2. Washing hands with soap and water more often than usual and using alcoholic hand gel more than usual | Mar. 2008 | **O** | $\boldsymbol{\times}$ | **O** | $\boldsymbol{\times}$ | $\boldsymbol{\times}$ |
| 3. Avoiding contact with birds and the excretion of birds | Mar. 2008 | $\boldsymbol{\times}$ | $\boldsymbol{\times}$ | $\boldsymbol{\times}$ | $\boldsymbol{\times}$ | $\boldsymbol{\times}$ |
| 4. Eating thoroughly cooked poultry and eggs | Mar. 2008 | **O** | **O** | $\boldsymbol{\times}$ | $\boldsymbol{\times}$ | $\boldsymbol{\times}$ |
| 5. Not buying uninspected animals and animal products | Mar. 2008 | **O** | $\boldsymbol{\times}$ | $\boldsymbol{\times}$ | **O** | $\boldsymbol{\times}$ |
