# Supplementary material for: People at Risk of Influenza Pandemics: The Evolution of Perception and Behavior
Source: PLoS One. 2015 Dec 14;10(12):e0144868. doi: 10.1371/journal.pone.0144868 (PMC4682843; doi:10.1371/journal.pone.0144868)
Supplement: S3 Table — (DOCX) [file pone.0144868.s003.docx]

**S3 Table. Responsive behavior among different demographic and socioeconomic groups (2009 surveys) (“O”: significant difference; “**$\boldsymbol{\times}$**”: no significant difference)**

|  |  | Gender | Age | Education | Self-reported health | Income |
| --- | --- | --- | --- | --- | --- | --- |
| 1. Ventilating living and working places | Aug. 2009 | $\boldsymbol{\times}$ | $\boldsymbol{\times}$ | $\boldsymbol{\times}$ | **O** | $\boldsymbol{\times}$ |
|  | Nov. 2009 | $\boldsymbol{\times}$ | **O** | $\boldsymbol{\times}$ | $\boldsymbol{\times}$ | $\boldsymbol{\times}$ |
| 1. Washing hands with soap and water more often than usual and using alcoholic hand gel more than usual | Aug. 2009 | **O** | **O** | $\boldsymbol{\times}$ | **O** | $\boldsymbol{\times}$ |
|  | Nov. 2009 | $\boldsymbol{\times}$ | **O** | **O** | **O** | $\boldsymbol{\times}$ |
| 1. Covering coughs and sneezes with paper tissues, handkerchief, or forearm | Aug. 2009 | $\boldsymbol{\times}$ | $\boldsymbol{\times}$ | $\boldsymbol{\times}$ | **O** | $\boldsymbol{\times}$ |
|  | Nov. 2009 | $\boldsymbol{\times}$ | **O** | **O** | **O** | $\boldsymbol{\times}$ |
| 1. Purchasing face masks and wearing them in hospitals and places | Aug. 2009 | $\boldsymbol{\times}$ | **O** | **O** | $\boldsymbol{\times}$ | $\boldsymbol{\times}$ |
|  | Nov. 2009 | **O** | **O** | $\boldsymbol{\times}$ | $\boldsymbol{\times}$ | **O** |
| 1. Staying away from places where many people gather, such as shopping malls | Aug. 2009 | $\boldsymbol{\times}$ | **O** | **O** | **O** | $\boldsymbol{\times}$ |
|  | Nov. 2009 | $\boldsymbol{\times}$ | **O** | $\boldsymbol{\times}$ | $\boldsymbol{\times}$ | $\boldsymbol{\times}$ |
| 1. Avoiding contact with people from infected areas | Aug. 2009 | **O** | $\boldsymbol{\times}$ | **O** | **O** | $\boldsymbol{\times}$ |
|  | Nov. 2009 | $\boldsymbol{\times}$ | **O** | $\boldsymbol{\times}$ | **O** | **O** |
| 1. Willing to stay at home or be quarantined for 7 days, once suspected of or confirmed with H1N1 flu | Aug. 2009 | $\boldsymbol{\times}$ | **O** | $\boldsymbol{\times}$ | **O** | $\boldsymbol{\times}$ |
|  | Nov. 2009 | $\boldsymbol{\times}$ | **O** | $\boldsymbol{\times}$ | $\boldsymbol{\times}$ | $\boldsymbol{\times}$ |
| 1. Talking with doctors or friends about health issues related to H1N1 or swine flu | Aug. 2009 | $\boldsymbol{\times}$ | **O** | **O** | $\boldsymbol{\times}$ | **O** |
|  | Nov. 2009 | **O** | **O** | $\boldsymbol{\times}$ | $\boldsymbol{\times}$ | $\boldsymbol{\times}$ |
| 1. Going to clinics or hospitals once having flu-like symptoms suspicious of the H1N1 flu | Aug. 2009 | $\boldsymbol{\times}$ | $\boldsymbol{\times}$ | $\boldsymbol{\times}$ | **O** | $\boldsymbol{\times}$ |
|  | Nov. 2009 | $\boldsymbol{\times}$ | **O** | $\boldsymbol{\times}$ | $\boldsymbol{\times}$ | $\boldsymbol{\times}$ |
| 1. Purchasing medicines for preventing and treating the flu such as Tamiflu or Relenza | Aug. 2009 | $\boldsymbol{\times}$ | **O** | $\boldsymbol{\times}$ | $\boldsymbol{\times}$ | $\boldsymbol{\times}$ |
|  | Nov. 2009 | $\boldsymbol{\times}$ | **O** | **O** | **O** | **O** |
| 1. Vaccinating for preventing seasonal flu | Aug. 2009 | **O** | **O** | **O** | $\boldsymbol{\times}$ | **O** |
|  | Nov. 2009 | $\boldsymbol{\times}$ | $\boldsymbol{\times}$ | **O** | **O** | $\boldsymbol{\times}$ |
| 1. Stockpiling food and water for two to four weeks at home | Aug. 2009 | **O** | $\boldsymbol{\times}$ | **O** | **O** | **O** |
|  | Nov. 2009 | $\boldsymbol{\times}$ | $\boldsymbol{\times}$ | $\boldsymbol{\times}$ | **O** | $\boldsymbol{\times}$ |
