# Supplementary material for: People at Risk of Influenza Pandemics: The Evolution of Perception and Behavior
Source: PLoS One. 2015 Dec 14;10(12):e0144868. doi: 10.1371/journal.pone.0144868 (PMC4682843; doi:10.1371/journal.pone.0144868)
Supplement: S1 File — (DOCX) [file pone.0144868.s004.docx]

# 北京市公众流感大流行风险认知调查问卷

## A 风险认知总体水平及风险防范准备情况

1. 您是否听说过流感大流行？（选1、2继续回答问题2，选3跳答问题4）

1 听说过且清楚它的意思 2 听说过但不清楚它的意思 3 从未听说过

1. 您认为流感大流行应是下列哪一项？（选4继续回答问题3，选其余答案的跳到问题4）

1 普通流感 2 禽流感

3 季节性流感 4 全球爆发或流行的病毒性人类流感

5 人感染禽流感病毒 6 其他（请注明）

1. 下面我们会提到一些有关流感大流行的信息，请问您是否知道这些信息？

（A）流感大流行病毒很可能来源于禽流感病毒的变异

1 知道 2 不知道

（B）流感大流行病毒主要通过空气实现远距离传播

1 知道 2 不知道

（C）一旦感染流感大流行病毒，开始时症状类似普通流感

1 知道 2 不知道

（D）病情发展迅速，易引起多种易致死的并发症

1 知道 2 不知道

1. 下面我们将向您介绍流感大流行的概念。流感大流行，是流感，但不同于普通流感，而是能导致全球爆发或流行严重疾病的病毒性人类流感，易于在人与人间传播，且人们很少有自然的免疫力。人类在20世纪共经受了3次大流感。听了这些介绍之后，您认为在未来12个月内，“流感大流行”暴发的可能性有多大？

1 非常可能 2 较可能 3 可能 4 较不可能 5 不可能 6 无法回答

1. 下面我们会提到一些流感大流行一旦暴发可能出现的情况，您觉得这些情况发生在您身上的可能性有多大？请您根据可能性大小进行打分，5分表示一定会发生，1分表示一定不会。

（A）自己被传染流感大流行

A 5 B 4 C 3 D 2 E 1 F 无法回答

（B）家人或亲密的朋友被传染流感大流行

A 5 B 4 C 3 D 2 E 1 F 无法回答（6）

（C）一旦自己被感染没有亲友可以照顾自己

A 5 B 4 C 3 D 2 E 1 F 无法回答（6）

（D）家庭面临经济困难

A 5 B 4 C 3 D 2 E 1 F 无法回答（6）

（E）买不到充足的药物

A 5 B 4 C 3 D 2 E 1 F 无法回答（6）

（F）得不到相应的医疗服务

A 5 B 4 C 3 D 2 E 1 F 无法回答（6）

1. 您家里是否常备下列的东西？

（A）您家中是否常备体温计？

1 是 2 否

（B）您家中是否常备治疗发烧的药物？

1 是 2 否

（C）您家中是否常备达菲？

1 是 2 否

1. 下面我们会提到一些做法，请问您现在是否已经做到？5分表示已经完全做到，1分表示完全没有做到。

（A）居室和工作场所经常开窗通风

A 5 B 4 C 3 D 2 E 1 F 无法回答（6）

（B）经常用肥皂和流水洗手

A 5 B 4 C 3 D 2 E 1 F 无法回答（6）

（C）减少与禽类接触，避免接触禽类的粪便和其他分泌物

A 5 B 4 C 3 D 2 E 1 F 无法回答（6）

（D）禽类、蛋类食物彻底煮熟再吃

A 5 B 4 C 3 D 2 E 1 F 无法回答（6）

（E）不购买和食用未经检疫的动物及制品

A 5 B 4 C 3 D 2 E 1 F 无法回答（6）

1. 请问您去年冬天是否注射了普通流感疫苗？（选1跳到问题10，选2继续答问题9）

1 是 2 否

1. 您没有注射普通流感疫苗的原因是什么？（可多选）

1 价格太贵 2 自己身体好没必要接种 3 对疫苗的安全性不放心

4 疫苗的保护效果有限 5 流感是小病得了没关系

6 不知道到什么地方接种 7 附近没有接种点 8 其他（请注明）____

1. 请问您得了普通流感后是否会去就医？

1 每次都去 2 经常会去 3 偶尔会去 4 从来不去

## B 影响风险认知水平的外部因素调查

1. 关于您的信息获取渠道，请您回答下列问题（可多选）

（A）在您经历过的传染病事件中（如SARS、禽流感等），关于传染病疫情的信息一般来自于哪里？

1 政府 2 专家 3 媒体上的非官方信息 4 周围人群 5 其他（请注明）

（B）关于疫苗和药物的信息一般来自于哪里？

1 政府 2 专家 3 媒体上的非官方信息 3 周围人群 5 其他（请注明）

1. 关于信息来源，请您回答下列问题（可多选）

（A）关于传染病疫情的信息来自于哪里您认为是可信的？

1 政府 2 专家 3 媒体上的非官方信息 4 周围人群 5 其他（请注明）

6 都不信

（B）关于疫苗和药物的信息来自于哪里您认为是可信的？

1 政府 2 专家 3 媒体上的非官方信息 4 周围人群 5 其他（请注明）

6 都不信

1. 下面我们会提出几个建议，请问这些建议来自哪里您会按它说的去做？

（A）“避免参与集体活动”的建议来自哪里您会照做？

1 政府 2 专家 3 媒体上的非官方的信息 3 周围人群 E 都不照做

（B）“减少出入公共场所”的建议来自哪里您会照做？

1 政府 2 专家 3 媒体上的非官方的信息 4 周围人群 E 都不照做

（C）“减少使用公共交通设施”的建议来自哪里您会照做？

1 政府 2 专家 3 媒体上的非官方的信息 4 周围人群 E 都不照做

（D）“减少食用禽类、蛋类等食品”的建议来自哪里您会照做？

1 政府 2 专家 3 媒体上的非官方的信息 4 周围人群 E 都不照做

1. 您平时是通过哪些途径了解传染病相关信息的？（可多选）

1 电视 2 广播 3 报刊、杂志 4 书籍 5 互联网 6 亲戚、朋友

7 录像制品和电子出版物 8 公交车电视、楼宇电视 9 街头宣传栏

10 商场、超市等公共场所的张贴 11 本单位组织的宣传演练

12 本社区组织的宣传演练 13 政府组织的宣传演练 14 学校开设课程讲座

15 医生护士 P 其他（请注明）_______________

1. 在以上所提到的途径当中，您认为哪一种最可信？（单选）

1 电视 2 广播 3 报刊、杂志 4 书籍 5 互联网 6 亲戚、朋友

7 录像制品和电子出版物 8 公交车电视、楼宇电视 9 街头宣传栏

10 商场、超市等公共场所的张贴 11 本单位组织的宣传演练

12 本社区组织的宣传演练 13 政府组织的宣传演练 14 学校开设课程讲座

15 医生护士 16 其他（请注明） 17 都不信

## H 个人基本信息

1. 总体上来说，您的健康状况是：

1 很差 2 差 3 一般 4 好 5 很好

1. 您在工作中是否直接与传染病患者接触？

1 是 2 否

1. 您在工作中是否直接与禽类接触？

1 是 2 否

1. 记录受访者的性别：

1 男 2 女

1. 请问您的最高学历是？

1 小学及以下 2 初中/技校 3 高中/中专 4 大专/大学

5 研究生及以上 6 不愿回答

1. 请问您今年的周岁年龄是____岁：

1. <18 2. 18-24 3. 25-29 4. 30-34 5. 35-39 6. 40-44

7. 45-49 8. 50-60 9. >=60 10. 不愿回答

1. 请问您在哪个区居住？

| 城八区 | | | | 远郊区县 | | | | | |
| --- | --- | --- | --- | --- | --- | --- | --- | --- | --- |
| 东城区 | A | 朝阳区 | E | 顺义区 | I | 房山区 | M | 密云县 | Q |
| 西城区 | B | 海淀区 | F | 昌平区 | J | 大兴区 | N | 延庆县 | R |
| 崇文区 | C | 丰台区 | G | 门头沟区 | K | 怀柔区 | O |  |  |
| 宣武区 | D | 石景山区 | H | 通州区 | L | 平谷区 | P |  |  |

1. 您的个人月收入水平大约相当于哪一个档次（包括年终分红及炒股等工资外的收入）？

1. ≤1000 2. 1001-2000 3. 2001-3000 4. 3001-4000

5. 4001-6000 6. 6001-8000 7. 8001-10000 8. ≥10000

9. 不愿回答
