# Supplementary material for: People at Risk of Influenza Pandemics: The Evolution of Perception and Behavior
Source: PLoS One. 2015 Dec 14;10(12):e0144868. doi: 10.1371/journal.pone.0144868 (PMC4682843; doi:10.1371/journal.pone.0144868)
Supplement: S2 File — (DOC) [file pone.0144868.s005.doc]

**甲型H1N1流感大流行期间**

**北京市市民的风险认知与行为选择调查**

**2009年8月**

**对甲型H1N1流感及其相关风险的基本认知**

1. 您是否知道“甲型H1N1流感”这个词语的含义？

A.知道含义 B.曾听说过，但并不知道它的含义 C.从没听说过

2. 就您所掌握的知识，通过以下哪种途径会感染及传播甲型H1N1流感？（可多选，并可以补充）？

A.在1米或2米之内的距离密切接触甲型H1N1流感患者

B.吃猪肉或与猪接触

C.直接接触患者的呼吸道分泌物或体液

D.患者打喷嚏或咳嗽时，将病毒传播给近距离接触者

E.其他途径，如__________

3. 据您了解，我国现在是否有可以预防目前流行的甲型H1N1流感的疫苗？

A.有，且储备充足

B.刚研发出来，还在人体试验阶段

C.没有

D.不知道

4. 据您了解，我国现在是否有能有效治疗甲型流感的药物？

A.有，且储备充足

B.有，但数量不多

C.没有

D.不知道

1. 您知道世界卫生组织已经将全球范围内甲型H1N1流感的疫情级别升级为第6级，即流感大流行阶段吗？

A.知道 B.不知道

1. 您认为能大流行的流感应是下面哪一项？
2. 普通感冒
3. 能在一定地区范围流行的季节性流感
4. 能在全球范围爆发或流行的人类新型流感
5. 人患高致病性H5N1禽流感 E 其他（请注明）：_______________________
6. 您是否知道甲型H1H1流感大流行在致病致死方面未来的严重化很可能因为该流感病毒将来突然变异或与其他流感病毒组合而来？

A.知道 B.不知道

1. 据您了解，您所在的区县、社区是否出现了甲型H1N1流感病例？

A.是的，已出现了病例 B.不，没有出现病例 C.不知道

1. 当今在世界范围甲型H1N1流感已经上升到流感大流行阶段，就您所掌握的信息，您认为以下这些情况发生的可能性有多大？（5分表示完全可能，1分表示完全不可能）

1）您本人感染甲型H1N1流感的可能性有多大？

A.非常可能 B.比较可能 C.有些可能 D.不太可能 E.根本不可能

F.无法回答

2）您的亲属或朋友感染甲型H1N1流感的可能性多大？

A.非常可能 B.比较可能 C.有些可能 D.不太可能 E.根本不可能

F.无法回答

3）甲型H1N1流感蔓延到您所在单位或社区的可能性有多大？

A.非常可能 B.比较可能 C.有些可能 D.不太可能 E.根本不可能

F.无法回答

4) 一旦该流感蔓延到您所在的单位或社区，您家庭将面临经济困难的可能性有多大？

A.非常可能 B.比较可能 C.有些可能 D.不太可能 E.根本不可能

F.无法回答

5）一旦该流感蔓延到您所在的单位或社区，您买不到充足药物的可能性有多大？

A.非常可能 B.比较可能 C.有些可能 D.不太可能 E.根本不可能

F.无法回答

6）一旦该流感蔓延到您所在的单位或社区，您得不到相应医疗服务的可能性有多大？

A.非常可能 B.比较可能 C.有些可能 D.不太可能 E.根本不可能

F.无法回答

1. 就全球而言，您认为甲型H1N1流感疫情还需要多长时间才可以得到有效控制？

A.2-3个月 B.半年左右 C.1年左右 D.更长时间 E.无法回答

**对甲型H1N1流感的行为选择**

1. 在现有甲型H1N1流感疫情条件下，您是否采取了以下个人防控措施？（您若有其他措施，也可以把它们补充进来）

|  | 是 | 否 |
| --- | --- | --- |
| a.尽量避免去人群聚集的场所，如体育比赛场馆、购物中心或公共交通场所等 |  |  |
| b.与医生或朋友交流有关甲型H1N1流感的健康话题。 |  |  |
| c.购买一定数量的口罩，去医院或人群聚集的场所能主动戴口罩 |  |  |
| d.咳嗽和打喷嚏时及时用手帕、纸巾或手臂捂住口鼻 |  |  |
| e.更经常地洗手或使用手部消毒用品，并避免用手直接接触口鼻眼等部位 |  |  |
| f.避免接触从疫区回来的人，特别是能避免接触流感样症状（发热，咳嗽，流涕等）或肺炎等呼吸道症状的病人 |  |  |
| g.室内尽量通风 |  |  |
| h.购买储备与流感防治相关的药物 |  |  |
| I．接种季节性流感疫苗 |  |  |
| J．若出现疑似症状，愿意及时主动去医院或卫生站接受检查 |  |  |
| K. 若出现疑似或确诊症状，愿意居家或在指定医院隔离治疗7天 |  |  |
| L. 在家中储备足够半个月到一个月的食品和饮用水 |  |  |
| M.其它，请补充：__________ |  |  |

1. 有关甲型H1N1流感疫苗

① 未来您是否愿意接种甲型H1N1流感疫苗？

A.愿意 B.不愿意 C.不知道

1. 如果您选择B，原因是什么？(可多选)

A价格太贵 B.自己身体好没有必要接种 C.对疫苗的安全性不放心

D.疫苗的保护效果有限 E.流感是小病得了没关系

F.不知道到什么地方接种 H.其他（请注明______）

**60周年国庆庆祝活动相关风险及政策选择**

1. 国庆庆祝活动期间您愿意参与大规模的群众活动吗？

A.非常愿意 B.比较愿意 C.不太愿意 D.根本不愿意 E.无法回答

1. 您担心国庆庆典这样大规模人群聚集活动会让北京甲型H1N1流感大流行的相关风险加大吗？

A.非常担心 B.有些担心 C.较不担心 D.根本不担心 E.无法回答

1. 您如果不担心，其原因是？（可以多选）

A.现阶段甲型H1N1流感病毒的致病、致死率不高，未来变异可能性小

B.我国政府的准备充分，现阶段防控措施较强，能够防患于未然

C.相信北京市政府的应急处置能力，即使疫情严重，市政府也能有效应对

D.其他（请注明）：_______________________________

1. 您如果担心，其原因是？（可以多选）
2. 甲型H1N1流感未来的致病、致死率可能提升，大规模集会会加大有关风险；
3. 我国相关药品和疫苗生产或储备不足，万一大范围传播，无法保障所有人的需求
4. 国庆庆典活动采取的防控措施还有漏洞，不能够防患于未然
5. 其他（请注明）:_____________________________________
6. 如果今年国庆前夕及国庆期间北京的甲型H1N1流感疫情出现或可能出现比较严重的局面（如大面积爆发或病死率升高），您认为国家和市政府应该采取以下哪项政策措施为好？

A．如期举行原定的各项庆典活动；

B. 提前或延后举行原定的各项庆典活动；

C．完全取消原定的各项庆典活动；

D. 举行非人群聚集的庆典活动，如电视广播庆典；网上庆典等

E. 采取其他合适的庆祝活动，并请列举：

**影响风险认知水平的外部因素调查**

1. 您是否经常关注各种媒体上关于甲型H1N1流感的信息？

A.非常关注 B.有一些关注 C.不太关注 D.根本不关注

1. 您获悉甲型H1N１流感相关信息的最主要渠道是什么（多选）：

A.电视 B.广播 C.报刊、杂志 D.书籍 E.互联网 F.亲戚朋友

G.手机短信 H.其他

（**访谈员一般不提示，但如果被访者回答不出来，可以适当提示**：如录像制品和电子出版物；公交车电视、楼宇电视；公益传单、街头宣传栏；商场、超市等公共场所的张贴物；政府、单位或者社区组织的宣传演练；学校开设的课程讲座；医护人员等）

1. 您知晓的关于甲型H1N1流感疫情和防控措施的信息一般来自于哪里？(可以多选)

A.政府 B.专家 C.媒体上的非官方信息 D.周围人群

E.其他（请注明 ）

1. 您认为来自哪方面的信息是可信的？

A.政府 B.专家 C.媒体上的非官方信息 D.周围人群

E.其他（请注明 ）

**个人基本情况**

**性别**：（1）男 （2）女

**健康状况：**（1）很好 （2）好 （3）一般 （4）差 （5）很差

（6）不知道

**年龄：**（1）18岁以下 （2）18-29岁 （3）30-39岁 （4）40-49岁

（5） 50-59岁 （6）60岁及以上

**教育程度：**（1）小学及以下 （2）初中 （3）高中/中专 （4）大学专科

（5）大学本科 （6）硕士及以上

**职业：**（1）学生 （2）进城务工农民 （3）企业职工 （4）事业单位职工

（5）国家机关工作人员 （6）农民 （7）个体劳动者 （8）离退休人员

（9）失业 （10）无职业 （11）其他

**收入水平（个人月收入）**：（1）≤1000 （2）1001－3000 （3）3001－5000

（4）5000以上

**来自哪个区县**：

来自：（1）城镇 （2）农村
