# Supplementary material for: People at Risk of Influenza Pandemics: The Evolution of Perception and Behavior
Source: PLoS One. 2015 Dec 14;10(12):e0144868. doi: 10.1371/journal.pone.0144868 (PMC4682843; doi:10.1371/journal.pone.0144868)
Supplement: S3 File — (DOCX) [file pone.0144868.s006.docx]

**甲型H1N1流感大流行期间**

**北京市市民的风险认知与行为选择调查**

**2009年11月**

**对甲型H1N1流感及其相关风险的基本认知**

1. 您是否知道“甲型H1N1流感”这个词语的含义？

1.知道含义 2.曾听说过，但并不知道它的含义 3.从没听说过

1. 就您所掌握的知识，通过以下哪种途径会感染及传播甲型H1N1流感？（可多选，并可以补充）

A. 在1米或2米之内的距离密切接触甲型H1N1流感患者

B. 吃猪肉或与猪接触

C. 直接接触患者的呼吸道分泌物或体液

D. 患者打喷嚏或咳嗽时，将病毒传播给近距离接触者

E. 其他途径

1. 据您了解，我国现在是否有可以预防目前流行的甲型H1N1流感的疫苗？

1.有，且储备充足 2.已经研发出来，通过临床试验，开始对重点人群接种

3.没有 4.不知道

1. 据您了解，我国现在是否有能有效治疗甲型流感的药物？

1.有，且储备充足 2.有，但数量不多 3.没有 4.不知道

1. 您认为能大流行的流感应是下面哪一项？

1.普通感冒 2.能在一定地区范围流行的季节性流感 3.能在全球范围爆发或流行的人类新型流感 4.人患高致病性H5N1禽流感 5.其他

1. 您是否知道甲型H1H1流感大流行在致病致死方面未来的严重化很可能因为该流感病毒将来突然变异或与其他流感病毒组合而来？

1.知道 2.不知道

1. 据您了解，现在北京市的甲型H1N1流感疫情的情况是:

1.出现聚集性爆发 2.有重症病例 3.出现死亡病例 4.疫情温和 5.不太了解

**对进入流感季节后疫情趋势的认知**

1. 当今在世界范围内，甲型H1N1流感已经上升到流感大流行阶段，进入秋冬流感季节后，您认为以下这些情况发生的可能性有多大？（5 非常可能 4 比较可能 3 有些可能 2 不太可能 1 根本不可能 6 无法回答）

1）您本人感染甲型H1N1流感的可能性有多大？

2）您的亲属或朋友感染甲型H1N1流感的可能性多大？

3）甲型H1N1流感蔓延到您所在单位或社区的可能性有多大？

4）一旦该流感蔓延到您所在的单位或社区，您家庭将面临经济困难的可能性有多大？

5）一旦该流感蔓延到您所在的单位或社区，您买不到您无法获得预防治疗甲型H1N1流感的药物的可能性有多大？

6）一旦该流感蔓延到您所在的单位或社区，您得不到相应医疗服务的可能性有多大？

1. 在北京进入秋冬流感季后，您担心甲型H1N1流感大流行的相关风险增加吗？

1.非常担心 2.有些担心 3.较不担心 4.根本不担心 5.无法回答

1. 您担心的原因有哪些?（第9题选择１、２的请回答本题，可多选）

A. 甲型H1N1流感未来的致病、致死率可能提升

B. 我国相关药品和疫苗生产或储备不足，万一大范围传播，无法保障所有人的需求

C. 政府及医院的应急能力不强

D. 正常生活和工作秩序受到影响

E. 其他【请注明】：_____________________________________

1. 您不感到担心的原因是？（第9题选择３、４的请回答本题，可多选）

A. 现阶段甲型H1N1流感病毒的致病、致死率不高，未来变异可能性小

B. 我国政府的准备充分，现阶段防控措施较强，能够防患于未然

C. 相信北京市政府的应急处置能力，即使疫情严重，市政府也能有效应对

D. 其他【请注明】：_____________________________________

**对甲型H1N1流感防控的行为选择**

1. 在现有甲型H1N1流感疫情条件下，您是否采取了以下个人防控措施？（您若有其他措施，也可以把它们补充进来）(1.是 0.否)

|  | 是 | 否 |
| --- | --- | --- |
| a.尽量避免去人群聚集的场所，如体育比赛场馆、购物中心或公共交通场所等 |  |  |
| b.与医生或朋友交流有关甲型H1N1流感的健康话题 |  |  |
| c.购买一定数量的口罩，去医院或人群聚集的场所能主动戴口罩 |  |  |
| d.咳嗽和打喷嚏时及时用手帕、纸巾或手臂捂住口鼻 |  |  |
| e.更经常地洗手或使用手部消毒用品，并避免用手直接接触口鼻眼等部位 |  |  |
| f.避免接触从疫区回来的人，特别是能避免接触流感样症状（发热，咳嗽，流涕等）或肺炎等呼吸道症状的病人 |  |  |
| g.室内尽量通风 |  |  |
| h.购买储备与流感防治相关的药物 |  |  |
| I．接种季节性流感疫苗 |  |  |
| J．若出现疑似症状，愿意及时主动去医院或卫生站接受检查 |  |  |
| K. 若出现疑似或确诊症状，愿意居家或在指定医院隔离治疗7天 |  |  |
| L. 在家中储备足够半个月到一个月的食品和饮用水 |  |  |
| M.其它，请补充 |  |  |

1. 有关甲型H1N1流感疫苗

① 未来您是否会去接种甲型H1N1流感疫苗？

A.一定会 B.不会 C.不确定是否会

② 如果您选择B或C，原因是什么？(多选)

A. 价格太贵

B. 自身没有感染甲型H1N1流感的重大风险，没必要接种

C. 对疫苗的安全性不放心

D. 疫苗的保护效果有限

E. 甲型H1N1流感得了也没关系

F. 不知道去什么地方接种

G. 不愿意打针

H. 已经注射了季节性流感疫苗，也可以起到预防作用

I. 不相信卫生部门官方宣称的甲流疫苗的安全性

J. 其他，请说明____________________

1. 请问您家中是否有小孩？

1.是 2.即将出生 3.否

1. 您是否会带孩子去接种甲型H1N1流感疫苗？

1.一定会 2.不确定是否会 3.不会

1. 若13选择B或C，且15题选择A, 请说明会带小孩接种疫苗的原因：

A. 小孩自身抵抗力弱

B. 小孩自我保护意识弱，没有采取足够的保护措施

C. 小孩所处的环境【如学校】更容易传播甲型H1N1流感

D. 其他，请说明__________________________

1. 如果您家中有小孩，您是否认为您孩子所在的学校在11月后需要尽快停课一至两周，以避免甲型流感在学校大面积爆发？

1.非常必要 2.有些必要 3.一般 4.不太有必要 5.完全没必要

1. 请问您认为是否有必要从部门、社区、单位、个人家庭等方面全方位防控流感疫情?

1.非常必要 2.有些必要 3.一般 4.不太有必要 5.完全没必要

**影响风险认知水平的外部因素调查**

1. 您是否经常关注各种媒体上关于甲型H1N1流感的信息？

A.非常关注 B.有一些关注 C.不太关注 D.根本不关注

1. 您获悉甲型H1N１流感相关信息的最主要渠道是什么？（可多选）

A.电视 B.广播 C.报刊、杂志 D.书籍 E.互联网 F.亲戚朋友 G.手机短信

H.其他

（访谈员一般不提示，但如果被访者回答不出来，可以适当提示：如录像制品和电子出版物；公交车电视、楼宇电视；公益传单、街头宣传栏；商场、超市等公共场所的张贴物；政府、单位或者社区组织的宣传演练；学校开设的课程讲座；医护人员等）

1. 您知晓的关于甲型H1N1流感疫情和防控措施的信息一般来自于哪里？(可多选)

A.政府 B.专家 C.媒体上的非官方信息 D.周围人群 E.其他（请注明）________

1. 您认为来自哪方面的信息是可信的？

A.政府 B.专家 C.媒体上的非官方信息 D.周围人群 E.其他（请注明）________

F.都不可信

1. 您认为最近各类媒体对于甲型H1N1流感的信息报导是否充足?

A.非常充足 B.比较充足 C.比较不足 D.严重缺乏 E.不知道

1. 您认为不够充足的信息有哪些方面？

A.北京市疫情情况

B.国内及国际疫情情况

C.国内外甲流防控的科研进展

D.我国政府抗击甲型H1N1流感所采取的各种政策和措施

E.其他国家政府抗击甲型H1N1流感所采取的措施

F.公民应当采取哪些自我防护措施的宣传

G.其他，请说明_____________

1. 您认为最近政府及各类媒体对于甲型H1N1流感的信息报导是否及时？

A.非常及时 B.比较及时 C.比较滞后 D.严重滞后 E.不知道

1. 您认为不够及时的信息有哪些方面？

A.北京市疫情情况

B.国内及国际疫情情况

C.国内外甲流防控的科研进展

D.我国政府抗击甲型H1N1流感所采取的各种政策和措施

E.其他国家政府抗击甲型H1N1流感所采取的措施

F.公民应当采取哪些自我防护措施的宣传

G.其他，请说明_____________

**对当前政府防控绩效的评价**

1. 您对于北京市政府到目前为止防控甲型H1N1流感的所采取的措施是否满意？

A.非常满意 B.比较满意 C.一般 D.比较不满意 E.非常不满

1. 您对于中央政府到目前为止防控甲型H1N1流感的所采取的措施是否满意?

A.非常满意 B.比较满意 C.一般 D.比较不满意 E.非常不满

1. 从目前的情况来看，请问您对政府控制疫情的信心是怎样的呢？

A.非常有信心 B.比较有信心 C.一般 D.不太有信心 E.根本没信心

**个人基本情况**

1.性别：（1）男 （2）女

2.健康状况：（1）很好 （2）好 （3）一般 （4）差 （5）很差 （6）不知道

3.年龄：（1）18岁以下 （2）18-29岁 （3）30-39岁 （4）40-49岁 （5）50-59岁 （6）60岁及以上

4.教育程度：（1）小学及以下 （2）初中 （3）高中/中专 （4）大学专科 （5）大学本科 （6）硕士及以上

5.职业：（1）学生 （2）进城务工农民 （3）企业职工 （4）事业单位职工 （5）国家机关工作人员 （6）农民 （7）个体劳动者 （8）离退休人员 （9）失业 （10）无职业 （11）其他

6.收入水平（个人月收入）：（1）≤1000 （2）1001－3000 （3）3001－5000 （4）5000以上

7.来自哪个区县：

8.来自：（1）城镇 （2）农村
